# Leveraging mice with diverse microbial exposures for advances in osteoimmunology

**DOI:** 10.3389/fendo.2023.1168552

**Published:** 2023-05-10

**Authors:** Sarah E. Little-Letsinger, Sara E. Hamilton

**Affiliations:** ^1^ Department of Evolutionary Anthropology, Duke University, Durham, NC, United States; ^2^ Department of Laboratory Medicine and Pathology, Center for Immunology, University of Minnesota, Minneapolis, MN, United States

**Keywords:** dirty mice, inflammatory bone loss, immune cells, skeletal gracilization, osteoporosis, bone homeostasis

## Abstract

The skeletal and immune systems are intricately intertwined within the bone marrow microenvironment, a field of study termed osteoimmunology. Osteoimmune interactions are key players in bone homeostasis and remodeling. Despite the critical role of the immune system in bone health, virtually all animal research in osteoimmunology, and more broadly bone biology, relies on organisms with naïve immune systems. Drawing on insights from osteoimmunology, evolutionary anthropology, and immunology, this perspective proposes the use of a novel translational model: the dirty mouse. Dirty mice, characterized by diverse exposures to commensal and pathogenic microbes, have mature immune systems comparable to adult humans, while the naïve immune system of specific-pathogen free mice is akin to a neonate. Investigation into the dirty mouse model will likely yield important insights in our understanding of bone diseases and disorders. A high benefit of this model is expected for diseases known to have a connection between overactivation of the immune system and negative bone outcomes, including aging and osteoporosis, rheumatoid arthritis, HIV/AIDS, obesity and diabetes, bone marrow metastases, and bone cancers.

## Historical perspectives into osteoimmunology

Osteoimmunology is a field of study concerning the interface between the skeletal and immune systems (1). Arron & Choi first coined this term in 2000 in a *Letter to Nature* discussing then-recently published work by Takayanagi et al. (1, 2) on T-cell mediated regulation of osteoclasts. At the time, the molecular mechanisms by which immune cells could regulate bone homeostasis had not yet been elucidated. In 1999, Wong et al. (3) discovered tumor necrosis factor-related activation-induced cytokine (TRANCE; also known as RANKL, OPGL) expressed on the surface of activated T cells stimulates osteoclast differentiation and activity. Wong et al.’s work led Arron & Choi to remark on an important observation: “T cells are working constantly to fight off the universe of foreign particles in which we live … What prevents these T cells from causing extensive bone loss?” (1, 3). Cue Takayanagi et al. – their 2000 *Letter to Nature* dissects a mechanism to prevent uncontrolled bone loss during inflammatory T-cell responses *via* IFNγ degradation of tumor necrosis factor receptor-associated factor 6 (TRAF6) in osteoclasts to prevent osteoclast differentiation and activity (2). Arron & Choi describe Takayanagi et al.’s work as “the answer”, but these studies, and the entirety of rodent research from the 1960s on, have relied on specific pathogen-free (SPF) mice with little to no exposure to this “universe of foreign particles.

A year prior to Arron & Choi and Takayanagi et al., Kung et al. (4) published a novel paradigm demonstrating T-cell-derived RANK is a crucial mediator of osteoclastogenesis in a rodent model of rheumatoid arthritis. Nearly a quarter of a century later, it is well established that T-cells are causal in inflammatory bone loss, including in HIV/AIDS, inflammatory bowel diseases, and osteoporosis in addition to rheumatoid arthritis (4–11). Indeed, the term ‘immunoporosis’ was recently coined in 2018 by Rupesh Srivastava and colleagues to highlight the importance of the immune system in the pathophysiology of osteoporosis (7). Immune cell activity is tightly linked to bone homeostasis and remodeling (12). This is perhaps best exemplified by osteoclasts, which are tissue-resident macrophages that resorb bone derived from the monocyte/macrophage lineage of hematopoietic stem cells. The monocyte/macrophage lineage is an instrumental component of the innate immune system, the first responders to invading pathogens and major producers of inflammatory cytokines.

Inflammatory cytokines are the communication molecules of the immune system – in other words, immune cell production, activation, and activity is coordinated through release and uptake of pro- and anti-inflammatory cytokines (13, 14). This means that production of inflammatory cytokines is a direct result of immune activation or detection of invading pathogens (13, 14). In relation to bone, there is an abundance of evidence describing inflammatory cytokines as key modulators, both positive and negative, of bone mass (15–29); interleukin (IL) 1, IL-6, IL-17, and TNFα are among the most commonly studied cytokines associated with inflammatory bone loss (7, 30, 31). Given the intertwining of the skeletal and immune systems, understanding and effectively modeling the immune system is critical for the study of osteoimmunology, and more broadly, bone biology.

## Gracilization of the modern human skeleton: insights from evolutionary anthropology

Modern humans have a more gracile, or slender, skeleton than earlier human ancestors and other apes. This gracility and the associated reductions in relative strength greatly increase the lifelong risk of osteoporosis (32). Skeletal gracilization has been documented in the cortical shaft of long bones (*e.g.*, Increased periosteal and endocortical expansion explain increased cortical thickness, cortical bone volume fraction, estimated polar moment of inertia; (33)) and in trabecular bone at metaphyses surrounding joints (34–37). Temporally, skeletal gracilization coincides with the Neolithic Revolution (33, 38), the transition from hunter-gatherer societies to agricultural communities (e.g. farming, domestication of livestock) beginning in approximately 10,000 BCE (see Lewis et al. (39) for an insightful and detailed explanation of pathogens and the Neolithic revolution). The Neolithic Revolution signifies an increase in infectious disease and pandemics globally. This rise in pathogen burden is attributed to sustained increases in population density, greater contact between humans and animals, and poor sanitary conditions.

Much of the literature surrounding skeletal gracilization posits increased sedentism, defined as living in a fixed residential location for a long period of time, in modern humans as the primary cause (see Madimenos (40) for a stellar review on the topic). According to the literature, increased sedentism implies an increase in sedentary behavior and thus reductions in biomechanical loading. While the transition from a nomadic lifestyle to one characterized by permanent dwellings certainly involved an increase in sedentism, an important distinction should be made that living in one place for a long period of time fails to inform physical activity levels, or biomechanical loading. We know today that agricultural communities, like the Shuar in Ecuador and Tsimane in Bolivia, have high rates of physical activity throughout their lives, as well as modern farmers (41–44). Exercise modes differentially impact skeletal geometry and microarchitecture (45, 46); here, differential loading patterns resulting from the transition from endurance-based distance running and walking to a more strength training-based agricultural labor is likely more explanatory than reduced physical activity levels. Indeed, the relative deficits in femoral strength but not humeral strength documented by Ruff and colleagues (38) may represent a change in habitual behavior resulting in greater mechanical loading of the upper limbs (i.e. through farming, carrying), rather than decreased loading of the lower limbs. Notably, femoral and humeral strength have stabilized in the past 2000 years (38), suggesting environmental and/or behavioral factors associated with the Neolithic revolution are relevant.

High physical activity levels, relatively frequent periods of extreme caloric restriction and/or starvation, and increased pathogen burden are likely all key factors contributing to skeletal gracilization in modern humans – and particularly to the acceleration in skeletal gracilization coinciding with the Neolithic Revolution. While outside the scope of this paper, diet composition and caloric intake are highly relevant to understanding these changes and can be generally conceptualized during the Neolithic Revolution as including a loss of diversity and quality, as well as increased vulnerability to food shortages and periods of starvation (39, 47). Nutrition (quantity, quality) is a crucial component of both immune function and bone homeostasis, representing an area of study that would benefit from deep investigation along the same context described here.

Broad support for the concept that pathogen burden contributed to skeletal gracilization can be found in *life history theory*. Life history theory posits that an unfavorable environment, like one with hostile factors (*e.g.* pathogens), leads to defense-oriented metabolic programming in which energy is prioritized for protective responses at the expense of growth and reproduction. Caloric intake and energy availability, particularly when coupled with physical activity (48, 49), are primary regulators of bone mass accrual and remodeling – even when omitting pathogen exposure as a variable. From an evolutionary standpoint, immune defense would likely be prioritized at the expense of skeletal growth and bone mass; this is particularly likely when considering bone structurally remodels in such a way to compensate for size-induced detriments in bone strength (50). Among the Shuar (forager-horticulturalists of Ecuador), children re-route energy expenditure from growth and development to immune defense during active infections (51). The Tsimane (forager-horticulturalists of Bolivia) demonstrate significant prevalence of low bone mineral density despite habitually high physical activity levels and an absence of risk factors common in industrialized societies (52, 53). The Tsimane demonstrate a greater incidence of osteoporosis risk (23% Shuar vs. 49% Tsimane vs. 34% US) and fracture (women: 18% Tsimane vs. 9% US; men: 36% Tsimane vs. 11% US) than the Shuar and US citizens (54, 55). Notably, both the Shuar and Tsimane maintain moderate to vigorous physical activity levels across the lifespan and the Tsimane have a higher pathogen burden than the Shuar or Americans (52–56).

## Embracing complexity: diverse microbial exposures as a basal feature of our environment

Our modern understanding of osteoimmune interactions and bone biology is built from studies conducted nearly exclusively with specific-pathogen free mice. To understand why this matters, let’s examine the work of Sjögren and colleagues (57) comparing conventionally raised and germ-free mice. Sjögren et al. demonstrated commensal (*i.e.*, friendly or non-pathogenic) microbiota act as key mediators of bone health through modulation of the immune system (8, 57, 58). Germ-free SPF mice exhibited significantly greater bone mass at 7 weeks of age than conventionally raised SPF mice (8, 57, 58). Bone mass was causally reduced following reconstitution of microbiota in germ-free mice through expansion of the T-cell compartment and increased levels of inflammatory cytokines (8). Jones et al. went on to demonstrate “lack of immune cell activation”, and thereby decreased immune cell crosstalk *via* inflammatory cytokines, as the cause of elevated bone mass in germ-free mice (58). The work of Sjögren, Jones, and their colleagues led to a burst in research centering on the role of the gut microbiome in bone health. Still, the physiologic impact of commensal and pathogenic organisms remains untested. Presumably, as pathogenic microbes initiate a vigorous host immune response, whereas commensal microbes do not, the impact of pathogenic exposure would cause a more robust reduction in bone mass, or a greater attenuation in bone mass accrual.

## Dirty mice as a novel translational model for osteoimmunology

Dirty mice (*e.g.*, wild or pet store mice) are characterized by diverse commensal and pathogenic microbial exposures. Such diverse exposures to the “universe of foreign particles” induces recurrent immune activation (59–61). Recurrent immune activation stimulates persistent inflammatory signaling resulting in sustained and elevated production of inflammatory cytokines that suppress bone formation and enhance resorption (62, 63). Dirty mice can be produced in a laboratory by several different methods, including co-housing, fomite bedding exposure from dirty mice, and natural microbiota transfer or rewilding – the strengths and weakness of different methods have been reviewed by Hamilton et al. (59). Regardless of generation method, immune activation can be confirmed by testing for a wide range of pathogens [see (59) for common pathogens in dirty mice generated by three different methods] and analysis of serum (*e.g.* flow cytometry, blood cell panel) to confirm expected immune changes in response to infection (64). Rosshart and colleagues have also demonstrated significant differences in the microbiome (gut, skin, and vagina), gut mycobiome, and gut virome of SPF and dirty mice generated using a wilding model, where SPF C57Bl/6 embryos are transplanted into wild mice transferring pathogen-free microbiota (65, 66). Here, we describe the key features distinguishing specific-pathogen free mice (SPF) and dirty mice, focusing on dirty mouse models transferring both commensal and pathogenic microbes, and discuss the relevance to the field of osteoimmunology.

### The immune system of specific-pathogen free mice closely matches human neonates

SPF mice maintain a high population of circulating naïve (~70%, CD62L^hi^CD44^lo^) CD8+ T cells and a low population of antigen-stimulated (~5%, CD62L^lo^CD44^hi^) CD8+ T cells (60). This immune profile is consistent with that found in human neonates but stands in contrast to adult humans. Pet store mice demonstrate an immune profile consistent with adult humans; specifically, a low population of circulating naïve (~19%, CD62L^hi^CD44^lo^) and a high population of antigen-stimulated (~47%, CD62L^lo^CD44^hi^) CD8+ T cells (60). Transfer of microbial exposures to SPF C57Bl/6 (B6) mice, *via* co-housing with pet store mice or their fomite bedding (64), induces sustained alterations to the basal immune profile like that seen in pet store mice and adult humans. While neither the total number of circulating immune cells nor the total number of CD4+ and CD8+ T cells differs between SPF and co-housed B6 mice, there are discrete differences in subtype, as in CD62L and CD44 expressing cells (60, 67). Co-housed B6 mice, in comparison to SPF B6 mice, exhibit elevations in circulating effector/effector memory CD4+ and CD8+ T cells, long-lived effector memory CD8+ T cells, monocytes, and neutrophils and reductions in B cells and natural killer cells (67).

In addition to circulating CD8+ T cells, Beura and colleagues demonstrate extensive changes to many innate and adaptive immune cell lineages in an array of tissues (60). To add further evidence, over 18,000 genes are differentially expressed between SPF and co-housed B6 mice; these data support gene expression profiles closely matching between immune naïve (*i.e.*, SPF mice and neonates) and immune experienced (*i.e.*, co-housed and pet store mice, adult humans) organisms (60). The transformation of the immune profile of the SPF B6 mouse following co-housing toward the profiles of pet store mice and adult humans, despite the differing genetic backgrounds, provides compelling causal evidence that immune system changes are due to environmental exposures.

### A mature immune system alters the basal inflammatory environment

**Figure 1:**
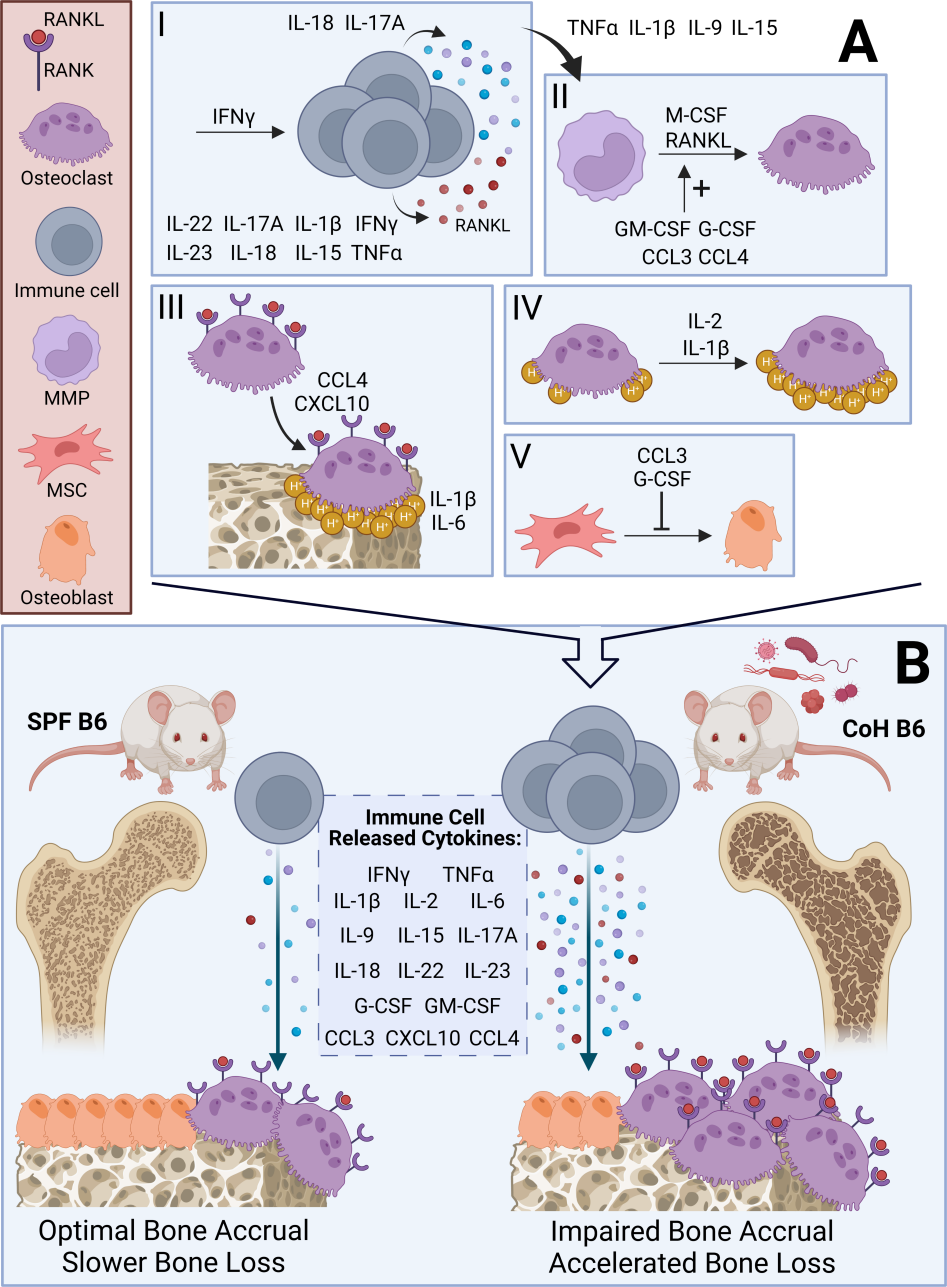
Hypothesized mechanisms and skeletal outcomes of osteoimmune interactions in SPF and Co-housed B6 mice. **(A)** Visual summary of effects of inflammatory cytokines outlined in **Table 1** on the differentiation and activity of osteoclasts and osteoblasts. I – IFNγ promotes immune cell activation. TNFα, IFNγ, IL-1β, IL-15, IL-17A, IL-18, IL-22, and IL-23 augment immune cell-dependent RANKL secretion. IL-17A and IL-18 upregulate immune cell-dependent secretion of inflammatory cytokines [see *Immune Cell Released Cytokines* in **(B)**]. II –TNFα, IL-1β, IL-9, and IL-15 enhance osteoclastogenesis induced by immune cell-dependent cytokines, including RANKL. GM-CSF, G-CSF, CCL3, and CCL4 increase differentiation of macrophage-monocyte progenitors (MMP) into osteoclasts, which requires M-CSF and RANKL. III – CCL4 and CXCL10 promote migration of osteoclasts to bone surfaces. At the bone surface, IL-1β and IL-6 promote bone resorption. IV – IL-1β and IL-2 increase acid (H^+^) production by osteoclasts. V – G-CSF and CCL3 inhibit mesenchymal stem cell (MSC) differentiation into osteoblasts. **(B)** Visual summary of effect of diverse microbial exposures in co-housed (CoH) B6 mice compared to SPF B6 mice. CoH B6 mice have an expanded T cell compartment and greater levels of immune cell-dependent inflammatory cytokines (*see Immune Cell Released Cytokines*). We expect this to impair bone mass accrual during adolescence and accelerate bone loss during aging leading to an osteoporotic phenotype *via* mechanisms outlined in **(A)**. We contrast CoH B6 mice with SPF B6 mice, which demonstrate a reduced T cell compartment and minimal production of inflammatory cytokines.

As we know, inflammatory cytokines are the communication molecules of the immune system; therefore, given the immune cell changes in co-housed B6 we could reasonably expect altered levels of inflammatory cytokines. Indeed, co-housed B6 mice demonstrate robust elevations in over 20 inflammatory cytokines (**Table 1**), even two months after microbial exposure (67). These inflammatory cytokines largely promote osteoclast differentiation and activity while suppressing osteoblast differentiation and activity, either directly or indirectly; though elevated circulating levels of IL-5, IL-10, IL-13, and CCL5 were also detected in co-housed and pet-store mice, with these cytokines inhibiting osteoclastogenesis (102) or promoting osteogenesis (103, 104). Still, such robust and comprehensive elevation in systemic inflammation is significant, particularly given that it is a basal environmental feature in dirty mice as it is in humans (105). Our scientific understanding of how complex cytokine profiles affect bone homeostasis in health and disease would benefit from utilization of the dirty mouse model (**Figure 1**).

**Table 1:** Inflammatory cytokines directly and indirectly stimulate bone resorption and suppress bone formation.

Cytokine	Effect of Cytokine on Bone
IFNγ	Indirectly stimulates bone resorption and bone loss *via* T-cell activation and secretion of RANKL* (15, 16)
TNFα	Increases RANKL expression and osteoclastogenesis (17–19), indirectly suppresses osteogenesis (20)
IL-1β	Enhances osteoclastogenesis, RANKL expression, and bone resorption (21, 22)
IL-2	Increases osteoclast acid production (68), though IL-2 deficiency leads to severe bone loss (69)
IL-6	Induces bone resorption (70, 71), involved in inflammatory bone loss, osteoporosis (23, 24)
IL-9	Enhances osteoclast number and activity, and implicated in rheumatoid arthritis (72, 73)
IL-15(R)	Increases osteoclast number and activity, upregulates RANKL expression, and implicated in rheumatoid arthritis, inflammatory bone loss (74–78)
IL-17A	Activates signaling cascades resulting in upregulation of TNFα, IL-1β, IL-6, RANKL, and M-CSF** (25–27)
IL-18	Enhances osteoclast number and activity, upregulates TNF and IL-6, and implicated in rheumatoid arthritis (79–81), but can indirectly inhibit osteoclastogenesis *via* osteoblast-derived GM-CSF (82)
IL-22	Induces osteoclastogenesis, upregulates RANKL expression, and implicated in rheumatoid arthritis (83, 84), but can indirectly stimulate osteogenesis (85)
IL-23	Increases RANKL expression and indirectly stimulates osteoclastogenesis *via* IL-17 (86–89)
G-CSF	Enhances osteoclastogenesis, indirectly suppresses osteogenesis, and implicated in osteoporosis (90–92)
GM-CSF	Extends the proliferation phase of pre-osteoclasts, enhancing osteoclastogenesis and bone resorption (28)
CCL3	Enhances osteoclastogenesis, suppress osteogenesis, and implicated in osteolytic lesions and aging-related osteoporosis (93–95)
CCL4	Promotes pre-osteoclast viability and migration to bone surfaces (96, 97)
CXCL10	Promotes osteoclast differentiation and migration to bone surfaces, and implicated in osteolytic lesions, osteoporosis, and rheumatoid arthritis (98–100)

*IFNγ directly stimulates anti-osteoclastogenic factors, but in infection the net balance is in favor of osteoclastogenesis (15).

**Macrophage colony stimulating factor (M-CSF) is requisite, with RANKL, for differentiation of osteoclasts (29, 101).

It is difficult to believe that the basal inflammatory environment in an organism with a mature immune system would not impair bone mass accrual prior to skeletal maturity and enhance bone resorption in aging. Even if no skeletal detriments occur from co-housing, this research will be valuable, potentially revealing novel osteoprotective therapeutic targets. In this event, one possible mechanism is in line with Takayanagi et al. (2): following T cell-mediated activation of the RANKL-TRAF6-NFκB-cSRC-JNK pathway that enhances osteoclast differentiation, activation, and survival, IFNy degrades TRAF6 within osteoclasts, preventing uncontrolled bone loss during inflammatory T-cell responses. Still, the work of Takayanagi et al. was performed in an organism with a naïve immune system, which we know demonstrates profound differences in gene expression and circulating immune cells and cytokines compared to an organism with a mature immune system. Much remains to be understood about the contributions of differing immune cell populations and immune cell-dependent inflammatory cytokine secretion in the context of bone biology and the bone marrow microenvironment. Experiments comparing bone homeostasis and remodeling between SPF and dirty mice are needed. Research areas linked to overactivation of the immune system, including aging and osteoporosis, rheumatoid arthritis, HIV/AIDS, obesity and diabetes, bone marrow metastases and bone cancers, will likely benefit from insights derived from the dirty mouse model.

## Conclusions

Adoption of the SPF model was intended to reduce the complexity of *in vivo* experimental systems, but the complexity arising from diverse microbial exposures isn’t noise. In the words of Maizels and Nussey, it “represents the genetic and environmental framework in which the immune system evolved and functions” (106). While the decades of experimental research using SPF mice have undoubtedly been valuable, it is time to embrace the complexity. Drawing on insights from osteoimmunology, evolutionary anthropology, and immunology in the above sections, we argue that the dirty mouse model will improve our understanding and treatment of bone loss caused by inflammation and immune activation. With rates of osteoporosis exceeding 1 in 3 women and 1 in 5 men globally, as well as the steep economic and health costs of an osteoporotic fracture, new therapeutic targets and interventions are needed (107). To identify these new targets, novel paradigms and models will need to be considered.

## Data availability statement

The original contributions presented in the study are included in the article/supplementary material. Further inquiries can be directed to the corresponding author.

## Author contributions

SL-L conceptualized and drafted the manuscript, table, and figure. SH critically evaluated and edited the manuscript, table, and figure. All authors contributed to the article and approved the submitted version.
